# miRNA mediated mitochondrial function and gene regulation associated with Alzheimer’s disease

**DOI:** 10.3389/fragi.2025.1582812

**Published:** 2025-05-13

**Authors:** Kumudu Subasinghe, Robert Barber, Nicole Phillips

**Affiliations:** ^1^ Department of Microbiology, Immunology, and Genetics, University of North Texas Health Science Center, Fort Worth, TX, United States; ^2^ Department of Family Health, University of North Texas Health Science Center, Fort Worth, TX, United States; ^3^ Institute for Translational Research, University of North Texas Health Science Center, Fort Worth, TX, United States

**Keywords:** Alzheimer’s disease (AD), mitochondrial function, microRNAs (miRNAs), neurodegeneration, gene regulation

## Abstract

MicroRNAs (miRNAs) are small non-coding RNA molecules that are known to regulate gene expression in their target locations thereby contributing to epigenetic mechanisms associated with disease pathologies. Dysregulation of miRNA activity has been implicated in the pathology of Alzheimer’s disease (AD), offering insights into potential biomarkers for early diagnosis and therapeutic targets. Mitochondrial dysfunction and its associated effects (such as oxidative stress) can be seen in early-onset AD. This review critically examines recent findings on mitochondrial-associated miRNAs—including miR-34a, miR-140, miR-455-3p, and miR-1273g-3p—highlighting their roles in mitochondrial bioenergetics, oxidative stress, and synaptic function. We discuss the therapeutic potential of targeting specific miRNAs to restore mitochondrial health and explore their utility as early biomarkers for AD diagnosis. A better understanding of miRNA-mediated mitochondrial regulation may open new avenues for early intervention in AD.

## 1 Introduction

Dementia has become a global health crisis according to the world health organization (WHO). Alzheimer’s disease (AD) is a predominant form of dementia that owns the similar urgency worldwide (Zhang et al., 2024). There are many risk factors associated with AD including genetics, epigenetics and environmental factors. Epigenetic modifications such as DNA methylation and RNA interference in gene expression are likely involved in many complex diseases including AD. Therefore, studying epigenetic mechanisms associated with disease pathologies is trending in research. Mitochondria provide energy necessary for cellular processes including neuronal cellular functions and synaptic activities. Disruption of mitochondrial function can lead to various disease pathologies and hence mitochondrial dysfunction is considered early hall mark for many diseases including AD.

**TABLE 1:** Summary of miRNA functions and their therapeutic potential in AD.

miRNA	Function in mitochondria	Role in AD	Therapeutic potential
miR-34a	Inhibits, ETC., gene expression	Promotes synaptic and mitochondrial dysfunction	Antagomir therapy
miR-455-3p	Promotes mitochondrial biogenesis	Neuroprotective, improves cognition in mice	Mimic and delivery show promise
miR-140	Targets PINK1, increases ROS	Induces apoptosis, worsens AD pathology	Potential target for inhibition
miR-1273g-3p	Reduces TIMM13 expression	Enhances Aβ production by inducing oxidative stress and mitochondrial impairment	Biomarker candidate for early AD

**FIGURE 1:**
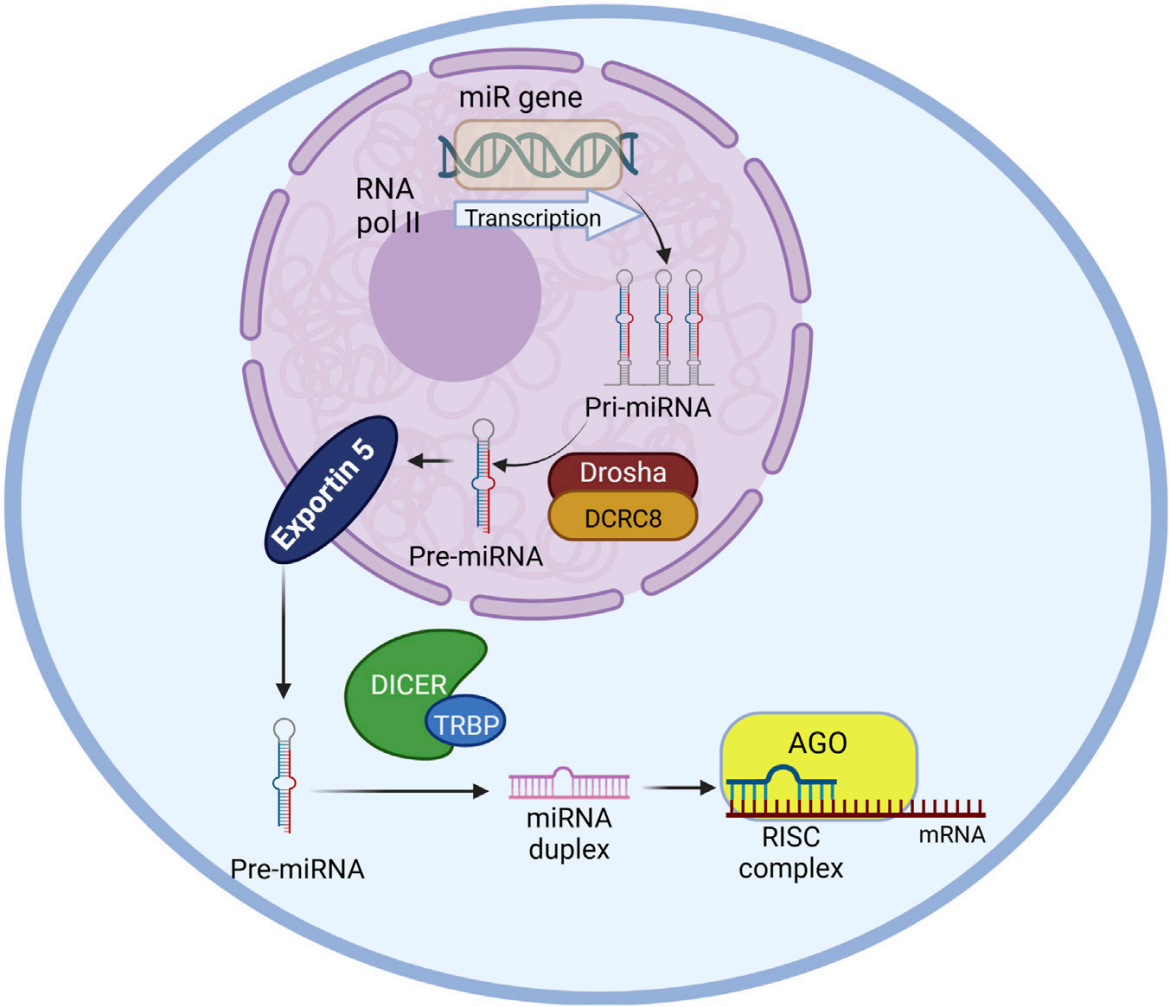
miRNA biogenesis pathway in a schematic diagram including primary enzymes and cellular compartments involved. The miR gene is transcribed by RNA polymerase II into pri-miRNA in the nucleus which will be further processed into pre-miRNA by Drosha-DCRC8 complex. The pre-miRNA will be transported to the cytoplasm by exportin five protein. The miRNA duplex will be generated from the pre-miRNA by the microprocessor-DICER-TRBP. Mature miRNA from the duplex will be selected by Ago protein and engaging together create the RNA-induced silencing complex (RISC) that induce target mRNA degradation. The image was created using biorender.com.

miRNAs play an important role in gene regulation of their target sites or organelles through their short complimentary seed sequence and preventing mRNAs from being translated. Recent studies indicate the association of several miRNA candidates in mitochondrial gene regulation, leading to mitochondrial dysfunction and AD pathology. Given the critical role of mitochondria in neuronal health, miRNAs that impair mitochondrial function may serve as early biomarkers or therapeutic targets in AD. This review critically examines the functional roles of mitochondrial-targeting miRNAs in AD pathogenesis and evaluates their diagnostic and therapeutic potential.

## 2 miRNA gene regulation mechanism

MicroRNAs (miRNAs) are small non-coding RNA molecules with about 18–22 nucleotides (Kumar and Reddy, 2020; Islam et al., 2024; Rivera et al., 2023) that are known to regulate gene expression in their target locations thereby contributing to epigenetic mechanisms associated with disease pathologies (Kumar et al., 2021). miRNA biogenesis (Figure 1) takes place in the nucleus by transcribing the gene by RNA polymerase II (Leitão and Enguita, 2022; Bartom et al., 2022). The resulting transcription product is a primary miRNA (pri-miRNA) precursor. These primary miRNA precursors are then processed by the Drosha/DGCR8 microprocessor complex by cleaving the primary miRNA precursor generating the precursor miRNAs (pre-miRNAs) using RNase III (Leitão and Enguita, 2022; Bartom et al., 2022). The pre-miRNA is then transported to the cytoplasm by Exportin-5. In the cytoplasm, the pre-miRNA is processed by the Dicer/TRBP complex by cleaving the pre-miRNA and generating mature miRNA which are dsRNA (double-stranded RNA) duplexes before they are loaded onto argonaute (Ago) proteins forming the RNA-induced silencing complex (RISC). The target mRNA recognition is done by the miRNA-induced silencing complex (miRISC) (Rivera et al., 2023) through Watson-Crick base pairing (Leitão and Enguita, 2022; Bartom et al., 2022).

miRNAs are associated with regulation of cell differentiation, cell cycle progression and apoptosis in many pathological processes including cancer, AD and neurodegeneration (Bartom et al., 2022; Jung et al., 2021). miRNA can induce gene silencing by targeting the 3′ untranslated region of the messenger RNA (mRNA) either by obstructing the translation or directing the mRNA for degradation (Islam et al., 2024; Bartom et al., 2022). The gene silencing activity involves only a very short region of complete complementarity between the ‘seed’, at position 2-7/8 of the guide strand of the miRNA. The ‘seed matches’ predominantly located in the 3′ untranslated region (3′ UTR) of targeted mRNAs (Bartom et al., 2022). This targeting results in gene silencing. Once synthesized in the nucleus, the mature miRNAs are transported to the cytoplasm and then transported towards their target location of tissues. Some miRNAs are reported to localize in their target organelles including mitochondria, nucleus, rough endoplasmic reticulum, processing (P)-bodies, early/late endosomes, multivesicular bodies, lysosomes and synaptosomes (Rivera et al., 2023). The miRNAs in the humans are involved in the processes associated with all development stages as well as pathological processes via regulating gene expression (Kumar and Reddy, 2020). It is known that a single miRNA can regulate multiple genes in their target locations (Sarkar et al., 2016).

## 3 Alzheimer’s disease

AD is a neurological disorder that progresses over time in the elderly population and is the primary cause of dementia. It is a heterogeneous complex disease with multiple causes including genetics, epigenetics, and environmental factors (Islam et al., 2024; Rivera et al., 2023). The aging population in the United States (US) is growing as well as the AD population. There are nearly six million individuals with AD-related dementia in the United States only and this number continues to rise with the increase of the aging population (Kumar and Reddy, 2020). Therefore, AD-related dementia has become a major burden to the healthcare system in the US economy as AD patents require extensive caregiving (Kumar and Reddy, 2020).

Accumulation of amyloid-β (Aβ) peptide in the form of amyloid plaques in the brain is considered an early event in AD. People with comorbidities such as diabetes, cardiovascular disease and hypertension have elevated risk of getting AD in their later stages of life. Dietary habits and the lifestyle also play major roles in risk for AD, which may function by increasing risk for AD-related comorbidities (Liang et al., 2021). Two indicators of AD are the buildup of amyloid plaques and intracellular neurofibrillary tangles of hyperphosphorylated tau protein. There are two major categories of AD based on the age of onset: early-onset AD (EOAD) and late-onset AD (LOAD) (Rivera et al., 2023; Wang et al., 2019). Given the multifactorial etiology and progressive nature of AD, current therapeutic strategies often fall short of addressing the underlying molecular mechanisms of the disease. This underscores the need for innovative approaches, such as miRNA-based therapies, which hold great promise in targeting the root causes of AD pathology.

## 4 Mitochondrial function in AD

Healthy mitochondrial function is important in brain health. Mitochondria are crucial for various essential functions in brain cells, particularly in neurons, where they supply synaptic energy (ATP), maintain Ca^2+^ balance, produce reactive oxygen species (ROS), regulate apoptosis, support mitophagy, facilitate axonal transport, and enable neurotransmission (Rivera et al., 2023). Mitochondrial dysfunction and its associated effects (such as oxidative stress) can be seen in early onset on AD. Recent studies show many mitochondrial abnormalities in AD brains (Rivera et al., 2023). These include disrupted mitochondrial bioenergetics, elevated oxidative stress, mitochondrial genomic damage, irregular mitochondrial fusion and fission, deficits in mitochondrial axonal transport, abnormal mitochondrial distribution, impaired mitochondrial biogenesis, dysfunctional endoplasmic reticulum–mitochondrial interactions, impaired mitophagy, and compromised mitochondrial proteostasis (Rivera et al., 2023). Mitochondrial dysfunction negatively affects the synaptic activities in AD (Rivera et al., 2023). Mitochondrial dysfunction’s central role in AD pathology highlights the critical need for targeted interventions aimed at restoring mitochondrial health. miRNA-based therapies present a particularly promising approach, given their ability to regulate multiple genes and pathways involved in mitochondrial function.

## 5 miRNAs associated AD risk through mitochondrial health

Synaptic activity is important in the ability to process information, forming memories and adapting to new environments. Therefore, synaptic dysfunction is a critical feature in AD that causes cognitive impairment and loss of neuronal function (Li et al., 2024). Mitochondria produce ATP necessary for synaptic signaling, hence, there is a significant correlation between the synaptic function and mitochondrial health. There are some miRNAs such as miR-484, miR-132, and miR-212 that are known to facilitate the release of neurotransmitters and maintain the integrity of the synapses (Li et al., 2024).

The APOE4 allele remains the most significant genetic risk factor for sporadic AD (Seyedaghamiri et al., 2023). Intriguingly, several studies have reported an inverse correlation between APOE4 presence and miR-195 expression in cerebrospinal fluid and brain tissue. This association suggests a mechanistic link where APOE4 may exacerbate mitochondrial dysfunction through miRNA-mediated pathways. Mini-Mental State Examination (MMSE) score indicates a negative correlation in cognitive performance with downregulation of miR-195. Importantly, overexpression of miR-195 in transgenic mouse models reduces hallmark AD pathologies—implying that modulating this miRNA could offer therapeutic benefit, particularly in APOE4 carriers. Similar association can also be seen with mir-107 in AD patients with APOE4 indicating its therapeutic advantage in APOE4 carriers (Li et al., 2024). Moreover, the studies done on mice with brain injuries show increase expression levels of miR-203, phosphorylated tau and APOE4. This expression levels were reduced by adding miR-203 inhibitors (Li et al., 2024).

## 6 miRNA in mitochondrial gene function

Mitochondrial localized miRNAs are known to impact mitochondrial gene regulation related to processes such as mitochondrial transport, calcium signaling, and synaptic vesicle formation (Li et al., 2024). Several recent studies indicate the role of some miRNA species that regulates the mitochondrial gene functions thereby associated with AD (Li et al., 2024; Kim et al., 2021). Many miRNAs are reportedly localized in the mitochondria that regulate local protein synthesis. Some of the miRNAs include miR-130a, miR-130b, miR-140, miR-320 and miR-494 and are enriched in the mitochondria as reported by Rivera et al., 2023 (Rivera et al., 2023). This localization implies a tightly regulated mechanism through which miRNAs orchestrate mitochondrial function at a post-transcriptional level. Such insights open potential avenues for targeted therapeutic interventions, particularly by modulating miRNA activity to restore mitochondrial integrity in disease states.

miRNAs not only affect mitochondrial function, but they also regulate oxidative stress. Gao et al., 2009 reports the connection of miR-23b in glutamine catabolism and disrupting the tricarboxylic acid (TCA) cycle resulting in reduction in ATP production (Gao et al., 2009). This disruption in energy metabolism could aggravate cellular vulnerability under stress conditions, implicating miR-23b as a potential biomarker for mitochondrial dysfunction. miR-210 has been reported to regulate mitochondrial function by upregulating glycolysis, activating generation of ROS, and ultimately causing mitochondrial dysfunction (Chen et al., 2010). Chen et al. further reports that miR-210 mainly target ISCU (iron-sulfur cluster scaffold homolog) and COX10 (cytochrome c oxidase assembly protein), two important factors of the mitochondria electron transport chain and TCA cycle (Chen et al., 2010). These findings emphasize miR-210’s dual role in energy metabolism and redox balance, suggesting that its dysregulation could be a pivotal event in mitochondrial pathologies. miR-338 is a brain specific miRNA that binds to nuclear-encoded mitochondrial mRNA coding for COXIV, hence plays a role in regulating axonal respiration and function by influencing the levels of COXIV, a protein essential for the assembly of the mitochondrial cytochrome c oxidase complex IV (Aschrafi et al., 2008). By modulating COXIV levels, miR-338 influences axonal respiration and function, highlighting its critical role in maintaining neuronal energy demands and synaptic activity. This underscores the importance of miRNA-mediated post-transcriptional regulation in neurodegenerative diseases, where disrupted mitochondrial function is a hallmark.

## 7 miRNA in AD-mitochondrial gene functions

### 7.1 miR-1273g-3p

Kim et al. studied miR-1273g-3p, which was reported to be expressed in cerebrospinal fluid (CSF) of early-stage AD patients. The study has used plasma and CSF of AD patients and healthy individuals to isolate miR-1273g-3p miRNA and analyze its interaction with targeted mitochondrial genes. The previous understanding of the function of miR-1273g-3p function is that with its overexpression, it can enhance the amyloid beta production by inducing oxidative stress and mitochondrial impairment. They used a biotin-streptavidin pull-down assay to demonstrate the miRNA interaction with the mitochondrial genes such as TIMM13 (translocase of inner mitochondrial membrane 13) which shows reduced expression in AD brains (Kim et al., 2021). TIMM13 combined with TIMM8p promotes the translocation of transmembrane space through binding to the membrane spanning domains in mitochondria which is essential for maintaining mitochondrial function and cellular homeostasis (Liao et al., 2023). TIMM13’s high expression in the brain underscores its importance in neuronal health and energy regulation, making its dysregulation a significant factor in neurodegenerative diseases.

The study’s findings not only illuminate the molecular underpinnings of miR-1273g-3p′s involvement in AD but also highlight its diagnostic potential. Its presence in peripheral samples such as CSF and plasma makes it an accessible and minimally invasive biomarker candidate for early AD detection. This is particularly significant given the need for reliable, early-stage diagnostic tools in AD, where therapeutic interventions could have a more pronounced effect. Furthermore, miR-1273g-3p′s role in promoting amyloid-beta production aligns with broader themes in AD research, linking oxidative stress and mitochondrial dysfunction to protein aggregation. This emphasizes the interconnectedness of molecular pathways in AD, where miRNA dysregulation could serve as both a trigger and a consequence of disease pathology.

### 7.2 miR-455–3P

microRNA-455–3P regulates mitochondrial gene expression and shows the potential to be a peripheral AD biomarker. Several studies report its protective effect against AD pathology and the mitochondrial biogenesis by promoting the upregulation of the PGC1α gene through the regulation of a novel signaling pathway called HIF1an-AMPK-PGC1α (Islam et al., 2024; Kumar et al., 2021). It is known that Mitochondrial structural and functional abnormalities play a central role in the progression of the disease and the disruption of synaptic function in AD. Kumar et al. report that a transgenic mice (TG) with miR-455-3p could live longer and contained improved cognitive behavior, spatial learning and memory compared to its aged wildtype. Further they report the reduction in mitochondrial function with the depletion of mir-455-3p by measuring the expression of genes associated with mitochondrial function: mitochondrial biogenesis (PGC1α, Nrf1, Nrf2, TFAM), mitochondrial dynamics (Drp1, Fis1, Opa1, Mfn1, Mfn2) and synaptic and dendritic (SNAP25, PSD95 and MAP2) genes using real-time qRT-PCR. The results indicate that miR-455-3p TG mice had better mitochondrial organization, as assessed by Transmission Electron Microscopy (TEM), which indicated improved mitochondrial quality. This improvement was corelated with the elevated levels of key mitochondrial biogenesis proteins, including PGC1α, NRF1, NRF2, and TFAM, which play essential roles in mitochondrial biogenesis. Additionally, the dynamics proteins involved in mitochondrial fission and fusion, such as DRP1, FIS1, OPA1, Mfn1, and Mfn2, were also upregulated in the TG mice, suggesting improved mitochondrial dynamics. Further, they discuss that HIF1α (Hypoxia-inducible factor 1-alpha) maybe regulated by miR-455-3p, which is known to influence mitochondrial function. The interaction between HIF1α and PGC1α plays a crucial role in regulating mitochondrial activity. Therefore, the study suggests that miR-455-3p, through its effects on HIF1α and PGC1α, may be a key regulator of mitochondrial function in TG mice (Kumar et al., 2021).

From a critical perspective, the findings by Kumar et al. underscore miR-455-3p′s multifaceted role in preserving mitochondrial integrity. The observed upregulation of biogenesis and dynamics-associated genes suggests that miR-455-3p not only enhances mitochondrial quantity but also improves their structural and functional quality. This dual action likely contributes to the observed cognitive and behavioral improvements in TG mice, positioning miR-455-3p as a therapeutic candidate for AD. However, the study raises questions about the specificity of miR-455-3p′s effects—are its benefits limited to AD, or might they extend to other neurodegenerative diseases characterized by mitochondrial dysfunction? Additionally, the proposed link between miR-455-3p, HIF1α, and PGC1α provides a compelling framework for understanding the molecular mechanisms underpinning mitochondrial regulation. Yet, the precise details of this interaction require further exploration, particularly in human models. Understanding the upstream signals that regulate miR-455-3p expression and its downstream effects on mitochondrial and neuronal health could provide deeper insights into its therapeutic potential.

### 7.3 miR-140

miR-140 has been reported to have associated with mitochondrial dysfunction and also reportedly enriched in the mitochondria (Rivera et al., 2023). Lian et al., 2021 reports their study on miR-140 directly target PINK1 gene and reduce its expression. PINK1 enhances mitochondrial function and cognition according to the previous studies. However, reduced expression of PINK1 leads to mitochondrial dysfunction, increases in the accumulation of mitochondrial ROS, and injury of the mitochondrial membrane, thereby induce neuronal cell apoptosis. This phenomenon has been observed in AD brains with increased expression of miR-140 indicating that miR-140 may have a causative effect in AD (Liang et al., 2021).

Critically, the study underscores the dual role of miR-140 as both a regulator and a disruptor of mitochondrial homeostasis. While its enrichment in mitochondria indicates a physiological role in regulating mitochondrial function under normal conditions, its overexpression in pathological states like AD highlights its potential contribution to disease progression. The direct targeting of PINK1 by miR-140 presents a mechanistic link between miRNA dysregulation and mitochondrial dysfunction, offering new insights into AD pathophysiology. From a broader perspective, the findings on miR-140 align with the growing recognition of mitochondrial dysfunction as a central feature of AD. The interplay between miRNAs like miR-140 and key mitochondrial regulators such as PINK1 emphasizes the importance of post-transcriptional regulation in maintaining mitochondrial integrity. The accumulation of mitochondrial ROS and subsequent neuronal apoptosis observed with increased miR-140 expression could amplify oxidative stress and inflammation in AD brains, creating a feedback loop that accelerates disease progression.

### 7.4 miR-34a

Sarkar et al. reported miR-34a having association with AD. MiR-34a is found to be overexpressed in the AD brain as well as AD mouse model. Sarkar et al. conducted an analysis of bioinformatics networks and pathways of the miR-34a target genes revealing that miR-34a can influence molecular processes closely associated with the regulation of pre- and post-synaptic neuronal excitability, mitochondrial oxidative phosphorylation, glycolysis, and resting-state functional connectivity (Sarkar et al., 2016). Their analysis further pointed to a reduced expression of a set of genes that are associated with the electron transport chain and mitochondrial function (e.g., NDUFC2, SDHC, UQCRB, UQCRQ, and COX10) as a result of increased expression of miR-34a, indicating its negative regulation of mitochondrial function (Sarkar et al., 2016).

miR-34a′s regulatory influence extends beyond mitochondrial function to neuronal excitability and synaptic connectivity (Payne et al., 2023; Fan et al., 2017). This underscores its central role in coordinating energy production with neural activity, processes that are tightly linked in the brain. The observed dysregulation of glycolysis and oxidative phosphorylation pathways further highlights the interconnectedness of cellular metabolism and neurodegeneration in AD. From a translational perspective, the repression of ETC-related genes by miR-34a links mitochondrial dysfunction directly to neuronal deficits in AD. This aligns with the broader understanding of AD as a disease of both metabolic and synaptic dysregulation. Future research could explore whether modulating miR-34a expression can restore mitochondrial function and improve neuronal connectivity, thereby offering a multi-faceted approach to combating AD.

## 8 Discussion

miR-34a, miR-140 and miR-1273g-3p appear to have toxic effect on mitochondrial function and could be early indicators of mitochondrial dysfunction whereas miR-455–3P has a protective effect against AD pathology. miR-455–3P could therefore be used as a therapeutic component in regaining mitochondrial function due to AD pathology. The measurement of the expression levels of miRNAs that are localized to mitochondria such as miR-130a, miR-130b, miR-140, miR-320 and miR-494 can also be important as their abnormal expression levels may be an indicator of mitochondrial dysregulation. miR-23b, miR-210 and miR-338 are also the regulators of mitochondrial functions and pathways that could be potential biomarkers.

### 8.1 Therapeutic targeting of mitochondrial miRNAs in AD

There are several translational Implications associated with these miRNAs including them as potential biomarkers. The stability of miRNAs in biofluids such as cerebrospinal fluid and blood highlights their potential as non-invasive biomarkers for early AD diagnosis. Comprehensive profiling of miRNA signatures—including miR-455-3p and miR-34a—may enable stratification of AD risk and monitoring of disease progression. As therapeutic targets, miRNA activity can be modulated utilizing several strategies including antagomirs, miRNA mimics and miRNA delivery approaches (Dai et al., 2024; Abdelaal et al., 2023). Antagomirs are synthetic chemically engineered antisense oligonucleotides that can silence specific miRNAs to prevent their activity. Antagomirs can be effective therapeutic strategy to hinder the activities of miR-34a, miR-140, miR-1273g-3p that show adverse association with mitochondrial function and AD pathology. miRNA mimics can restore the function of downregulated miRNAs such as miR-455-3p that shows neuroprotection against AD. Nanoparticle-based miRNA delivery mechanism can also be utilized in such miRNAs ensuring specificity and minimizing off-target effects. Lipid-based nanoparticles, exosome-mediated delivery are novel approaches that are being under investigation. CRISPR-based miRNA editing are also being actively explored to overcome these hurdles (Hong et al., 2020) (Table 1).

### 8.2 Critical analysis and limitations

While substantial progress has been made in elucidating miRNA-mediated mitochondrial dysfunction, several challenges remain. The redundancy and pleiotropy of miRNAs necessitate a careful balance between therapeutic efficacy and potential side effects. Furthermore, variability in miRNA expression across individuals and disease stages complicates biomarker development. Integrative multi-omics approaches and longitudinal studies will be essential to address these limitations. Translational efforts must prioritize the identification of miRNAs with dual roles as biomarkers and therapeutic targets. For instance, miR-34a′s well-documented involvement in mitochondrial dysfunction underscores its promise but also necessitates further validation in clinical settings (Seyedaghamiri et al., 2023; Payne et al., 2023; Dai et al., 2024).

## 9 Conclusion

Mitochondrial dysfunction is a pivotal event in the early stages of AD, and miRNAs have emerged as critical regulators of mitochondrial gene expression. This review highlights both pathological (miR-34a, miR-140, miR-1273g-3p) and protective (miR-455-3p) miRNAs with established roles in mitochondrial health and AD progression. These miRNAs not only reflect underlying disease processes but also represent viable targets for therapeutic modulation. Future studies should prioritize *in vivo* validation of miRNA functions, development of targeted delivery systems, and integration of miRNA biomarkers into clinical diagnostic pipelines. Advancing our understanding of miRNA–mitochondria crosstalk may ultimately lead to more effective strategies for early AD diagnosis and intervention.
